# High speed, long range, deep penetration swept source OCT for structural and angiographic imaging of the anterior eye

**DOI:** 10.1038/s41598-022-04784-0

**Published:** 2022-01-19

**Authors:** Siyu Chen, Benjamin Potsaid, Yan Li, Junhong Lin, Yunchan Hwang, Eric M. Moult, Jason Zhang, David Huang, James G. Fujimoto

**Affiliations:** 1 Department of Electrical Engineering and Computer Science, Research Laboratory of Electronics, Massachusetts Institute of Technology, Cambridge, MA 02139 USA; 2 Advanced Imaging Group, Thorlabs Inc., Newton, NJ 07860 USA; 3 Casey Eye Institute, The Center for Ophthalmic Optics and Lasers, Oregon Health and Science University, Portland, OR 97239 USA; 4 Health Sciences and Technology, Harvard and Massachusetts Institute of Technology, Cambridge, MA USA

**Keywords:** Translational research, Medical imaging, Eye diseases

## Abstract

This study reports the development of prototype swept-source optical coherence tomography (SS-OCT) technology for imaging the anterior eye. Advances in vertical-cavity surface-emitting laser (VCSEL) light sources, signal processing, optics and mechanical designs, enable a unique combination of high speed, long range, and deep penetration that addresses the challenges of anterior eye imaging. We demonstrate SS-OCT with a 325 kHz A-scan rate, 12.2 µm axial resolution (in air), and 15.5 mm depth range (in air) at 1310 nm wavelength. The ultrahigh 325 kHz A-scan rate not only facilitates biometry measurements by minimizing acquisition time and thus reducing motion, but also enables volumetric OCT for comprehensive structural analysis and OCT angiography (OCTA) for visualizing vasculature. The 15.5 mm (~ 11.6 mm in tissue) depth range spans all optical surfaces from the anterior cornea to the posterior lens capsule. The 1310 nm wavelength range enables structural OCT and OCTA deep in the sclera and through the iris. Achieving high speed and long range requires linearizing the VCSEL wavenumber sweep to efficiently utilize analog-to-digital conversion bandwidth. Dual channel recording of the OCT and calibration interferometer fringe signals, as well as sweep to sweep wavenumber compensation, is used to achieve invariant 12.2 µm (~ 9.1 µm in tissue) axial resolution and optimum point spread function throughout the depth range. Dynamic focusing using a tunable liquid lens extends the effective depth of field while preserving the lateral resolution. Improved optical and mechanical design, including parallax “split view” iris cameras and stable, ergonomic patient interface, facilitates accurate instrument positioning, reduces patient motion, and leads to improved imaging data yield and measurement accuracy. We present structural and angiographic OCT images of the anterior eye, demonstrating the unique imaging capabilities using representative scanning protocols which may be relevant to future research and clinical applications.

## Introduction

Optical coherence tomography (OCT) and OCT angiography (OCTA) have become indispensable in ophthalmic clinics, imaging depth-resolved structure and microvasculature with exquisite detail^1–4^. Anterior eye OCT imaging was demonstrated shortly after the invention of OCT^5^. Recent clinical applications, including anterior ocular biometry, investigating corneal and lenticular pathologies, angle assessment in glaucoma, and OCTA for the anterior segment vasculature, spurred the development of OCT technologies designed specifically for the anterior eye^6–9^.

The requirements and challenges for anterior eye OCT imaging differ from its retinal counterpart. Anatomically, the anterior segment extends from the corneal surface to the lens capsule, or approximately 8 mm in normal adult eyes. Some anterior eye tissues, such as the iris and sclera, are highly turbid and limit imaging to more superficial layers. To cover the entire anterior eye range, achieve sufficient penetration, or reach the scan speed required for OCTA, commercial and earlier prototype instruments often tradeoff imaging resolution, speed, range, and/or functionality^9^. Anterior eye imaging requires accurate positioning and detection of eye movements. Misalignment and motion can cause errors in biometry, as well as introduce parasitic noise in OCTA.

This study reports the development of swept-source OCT (SS-OCT) technology that combines advancements in light source, optics, mechanical design, and signal processing to address the challenges of anterior eye imaging. The unique combination of high 325 kHz A-scan speed, long 15.5 mm imaging range, dynamic focal plane adjustment, and versatile wide field OCT and OCTA imaging represents significant improvements over existing instruments. The prototype SS-OCT instrument uses a microelectromechanically actuated, wavelength tunable, vertical-cavity surface-emitting laser (MEMS-VCSEL) operating in the 1310 nm wavelength range. Compared to the shorter wavelengths used in retinal imaging, the 1310 nm range has reduced tissue scattering and allows deeper penetration into the iris, sclera, and anterior chamber angle^10^. The MEMS-VCSEL is optimized to scan wavenumber (*k*), or optical frequency, linearly in time^11^, allowing efficient use of analog-to-digital (A/D) conversion bandwidth. Dual channel acquisition of the OCT fringe signal as well as a Mach Zehnder interferometer (MZI) enables sweep to sweep *k* calibration, which corrects for variations in the MEMS-VCSEL *k* sweep, dispersion imbalance, and detector amplitude and phase variation with frequency^12,13^. This enables the SS-OCT instrument to achieve a constant axial resolution of 12.2 µm (~ 9.1 um in tissue) and optimum OCT point spread function (PSF), as well as a sensitivity of 105 dB over most of the 15.5 mm (~ 11.6 mm in tissue) depth range. High speed, focus tunable liquid lens technology extends effective depth focal range without sacrificing transverse resolution^14,15^. To facilitate clinical imaging and improve measurement repeatability, two angled iris cameras with a split parallax view are used to align both the transverse and axial instrument position to the pupil of the eye. The ergonomic patient interface allows rapid instrument alignment and provides good stability against vibration and patient head motion. Combining these advances enables simultaneous high speed, long range, deep penetration OCT and OCTA imaging using a single, integrated platform which will facilitate future clinical studies. This study presents representative OCT and OCTA imaging results of the anterior eye using example scanning protocols which demonstrate the unique capabilities of the SS-OCT technology and suggest possible future research and clinical applications.

## Results

### OCT imaging performance

A key requirement for long range imaging and accurate biometry is that the axial resolution and sensitivity should not vary over the imaging range. This requirement was achieved by using SS-OCT with an optimized MEMS-VCSEL light source with a linear wavenumber (*k*) sweep, dual channel acquisition of the OCT fringe and MZI calibration signals, and sweep to sweep *k* calibration^11–13^.

**Figure 1:**
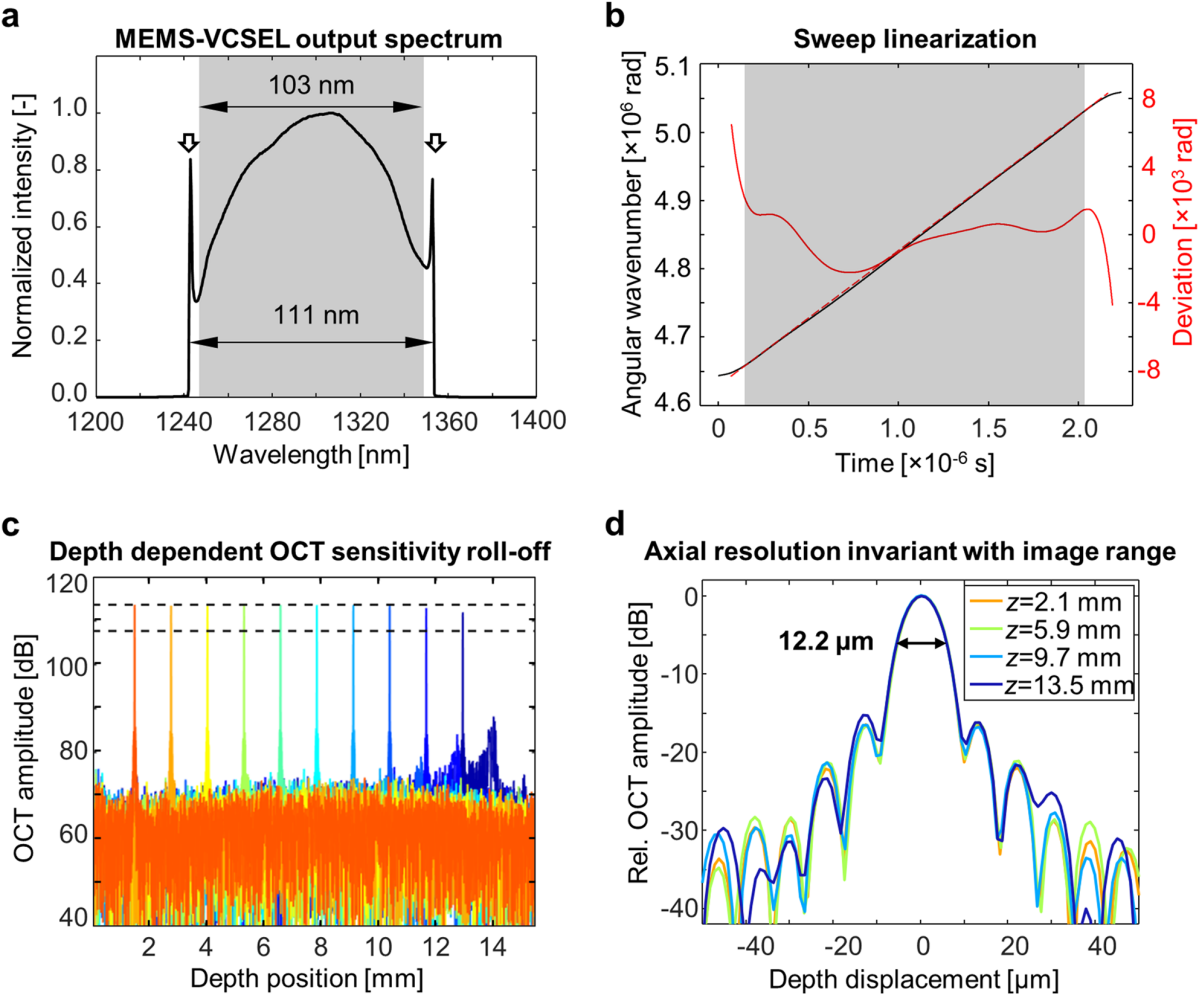
SS-OCT imaging performance. (**a**) Time-integrated spectrum of the MEMS-VCSEL light source. The booster optical amplifier driving current is modulated to achieve a symmetrical bell-shaped spectrum for improved sensitivity and sidelobe suppression. Arrows: spikes in spectral density occur because of the slowdown and reversal of the wavelength sweep. (**b**) The MEMS-VCSEL wavelength sweep with respect to time. The solid red line indicates difference between the measured sweep wavenumber (black) and ideal linear sweep (red dashed line). Shaded area represents the wavelength band used in OCT signal reconstruction. (**c**) OCT sensitivity versus imaging depth. Note that a constant sensitivity is maintained within the first ~ 13.5 mm axial range. Dashed lines correspond to a 6 dB drop in sensitivity. (**d**) OCT point spread functions from 4 imaging depths. The axial resolution is 12.2 µm full-width at half-maximum (FWHM) in air, and is invariant for imaging depths <  ~ 13.5 mm.

The output of the MEMS-VCSEL (Fig. 1a) spans a wavelength range of 111 nm centered around ~ 1310 nm, measured by an optical spectral analyzer (AQ-6315E, Ando Electric Co.). The output spectrum is shaped by modulating the current to the booster optical amplifier^16,17^. Because the laser safety exposure limit^18^ constrains the total allowable power delivered to the eye, spectral shaping improves imaging sensitivity by allocating more energy to the center, linearized portion of the sweep. A symmetric and bell-shaped spectrum also suppresses sidelobes of the OCT PSF, analogous to the application of a window function. Excluding the edges of the spectrum, which correspond to the slow down and reversal of the wavelength sweep, the usable bandwidth for OCT imaging is 103 nm. The instantaneous phase of the MEMS-VCSEL sweep is measured using the calibration MZI, and the difference from the ideal linear *k* sweep is calculated as shown in Fig. 1b. The relative phase error is kept below 0.08% within the OCT imaging wavelength band.

The instrument sensitivity and axial resolution are characterized by placing a mirror and neutral density filter (NENIR20A, Thorlabs Inc., measured OD = 2.47 at the illumination wavelength) within the OCT sample arm. All values are measured in air (refractive index *n* =  ~ 1.0). The peak sensitivity of the prototype SS-OCT instrument is 105 dB (roundtrip optical attenuation plus ratio of signal to the mean + standard deviation of background) with 5 mW incident power, which conforms to the latest American National Standards Institute (ANSI) laser safety exposure limit^18^. The OCT PSFs at different imaging depths are plotted in Fig. 1c. There is essentially no sensitivity roll-off within the first ~ 13.5 mm of the 15.5 mm imaging range. At deeper locations, the limited electronics bandwidth and aliasing caused by residual sweep non-linearity lead to a small reduction in OCT sensitivity and the appearance of a broadened secondary peak ~ 25 dB below the PSF peak.

Figure 1d shows enlarged axial PSFs at selected depth locations. These PSFs were reconstructed without applying the window function on the OCT interference fringe in order to assess consistency with the MEMS-VCSEL spectral sweep. The width of the PSF center lobe and the sidelobe levels are consistent with the Fourier transform of the resampled spectrum, thus confirming optimum *k* calibration and phase distortion correction. The full-width-at-half-maximum (FWHM) of the main PSF peaks are 12.2 µm in air. This axial resolution is invariant up to an imaging range of ~ 13.5 mm. The lateral resolution is ~ 25.0 µm using the 1/e^2^ intensity of the focused spot size. The depth of focus is ~ 756 um, defined as the confocal parameter or two times the Rayleigh range.

### Dual Iris camera guided alignment and structural imaging of the cornea

One important application of anterior eye OCT imaging is to measure the topography of the cornea, i.e., its curvature and thickness^19,20^. For this purpose, radial meridian scans, consisting of linear B-scans intersecting the apex of the cornea, are often used. Relying on the near circular symmetry of the cornea, radial scans require fewer A-scans compared to a full raster scan, markedly reducing the acquisition time and mitigating the effects of eye motion. However, lateral translations of the eye break the symmetry and cause measurement errors. Thus, aligning the radial “pivot” point precisely with the center of the cornea is critical for accurate and repeatable measurements. The use of dual iris cameras with a parallax “split view,” in addition to OCT pilot scans, can facilitate instrument alignment and reduce positioning error. High speed decreases the acquisition time, which additionally reduces the effects of involuntary eye movement.

**Figure 2:**
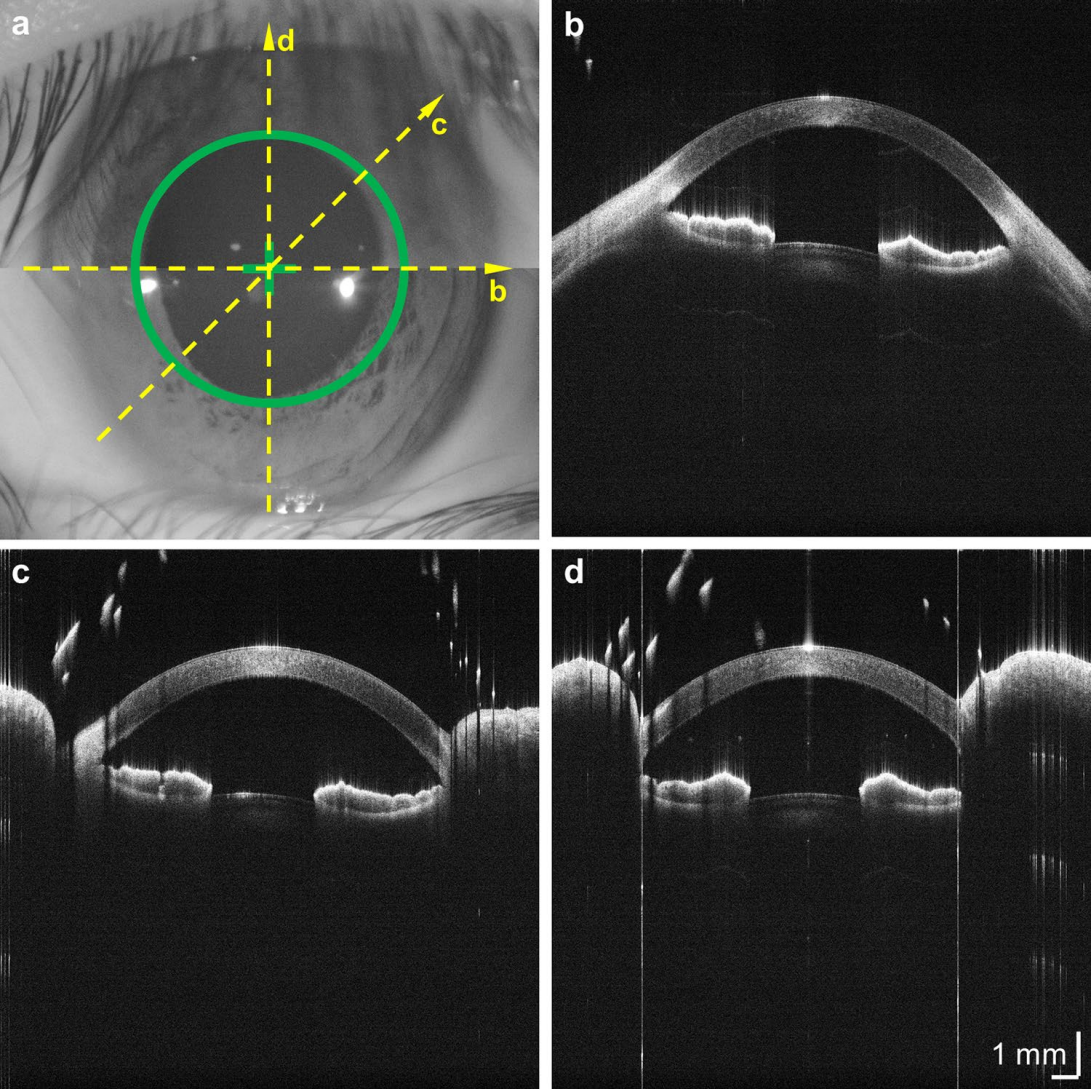
Split view iris camera view and radial corneal-scleral scans. (**a**) Composite “split view” iris image after optimum instrument alignment. Iterative adjustments in the transverse and axial direction center the image and align the top and bottom halves, placing the iris in the center of the OCT scan field and at correct axial working distance. (**b**)–(**d**) 20-mm OCT B-scans from 3 meridians indicated in (**a**), respectively. Accurate alignment is especially important for biometry applications.

The positioning accuracy was tested by repeatedly aligning the SS-OCT instrument 5 times and imaging a fixed target with a 3 mm circular center hole, representing the human pupil. The instrument operator used only the real-time video from the iris split view camera during the alignment. The acquired OCT volume was used as the reference for the true position. The standard deviation of the lateral and axial positioning error was 53 µm and 52 µm, respectively. The measured value for lateral positioning error is comparable to the SS-OCT transverse image resolution. Thus, the expected impact on measurement accuracy and repeatability should be small. However, practical positioning accuracy in the clinic will depend on operator skill and patient compliance. The video record of the iris cameras, synchronized to OCT acquisition at about 45 frames per second, can be used to verify correct eye positioning, detect eye movements, and reject invalid scans after acquisition. Figure 2 shows a representative split view camera image of a correctly aligned iris, as well as 20-mm B-scans with horizontal, diagonal, and vertical orientations from a healthy subject. Supplementary Video V1 shows the composite split view of the iris during instrument alignment.

**Figure 3:**
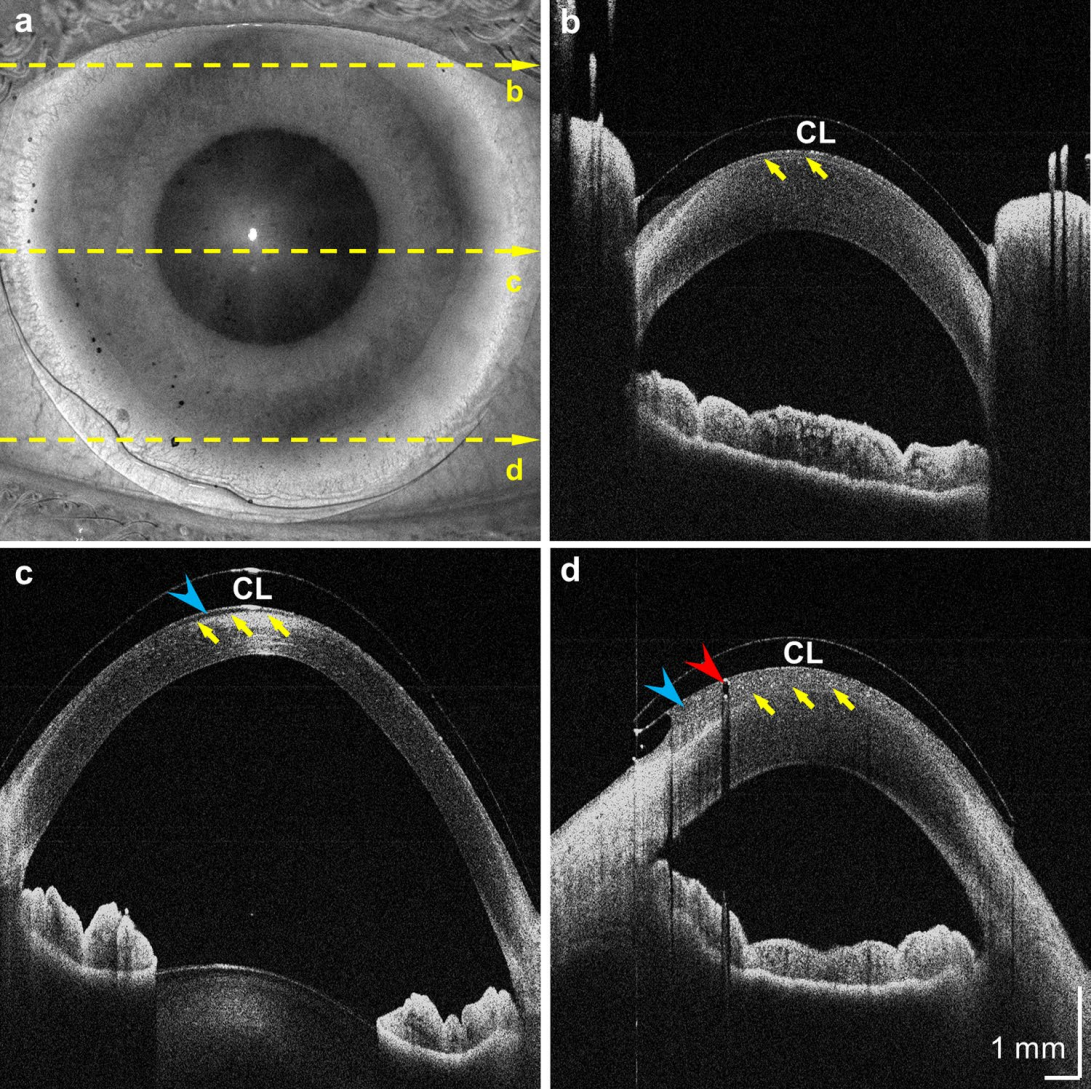
15-mm OCT volume of an eye with a hard contact lens (CL). (**a**) *En face* projection of the OCT signal. (**b**)–(**d**) B-scans extracted from locations indicated in (**a**). Yellow arrows: corneal epithelium. Blue arrowhead: fluid trapped between the contact lens and the cornea. Red arrowhead: Air bubble inclusion. The sharp discontinuity in the refractive index creates a strong shadowing artifact.

The fast 325 kHz A-scan rate of the SS-OCT prototype also enables raster scans of a square field of view covering the entire cornea. Figure 3 shows an example of 15-mm structural OCT imaging of an anterior eye with a hard contact lens. The contact lens appears as a hypo-scattering layer, with hyper-scattering interfaces between air and tear film. The tear film is moderately scattering. The tear film and corneal epithelium interface can be visualized, but the contrast is lower due to similar refractive indices. Notably, the fluid reservoir between the contact lens and cornea is more prominent on the lower-left corner, indicating a curvature mismatch (Fig. 3d, blue arrowhead). This asymmetric non-conformity suggests that raster scans, with evenly spaced A-scan sampling, can be beneficial in assessing corneoscleral surface. Indeed, the assumption of circular symmetry is progressively invalidated as the imaging field of view extends beyond the cornea^21,22^.

### Long range, deep penetration imaging of the corneoscleral and entire anterior segment

**Figure 4:**
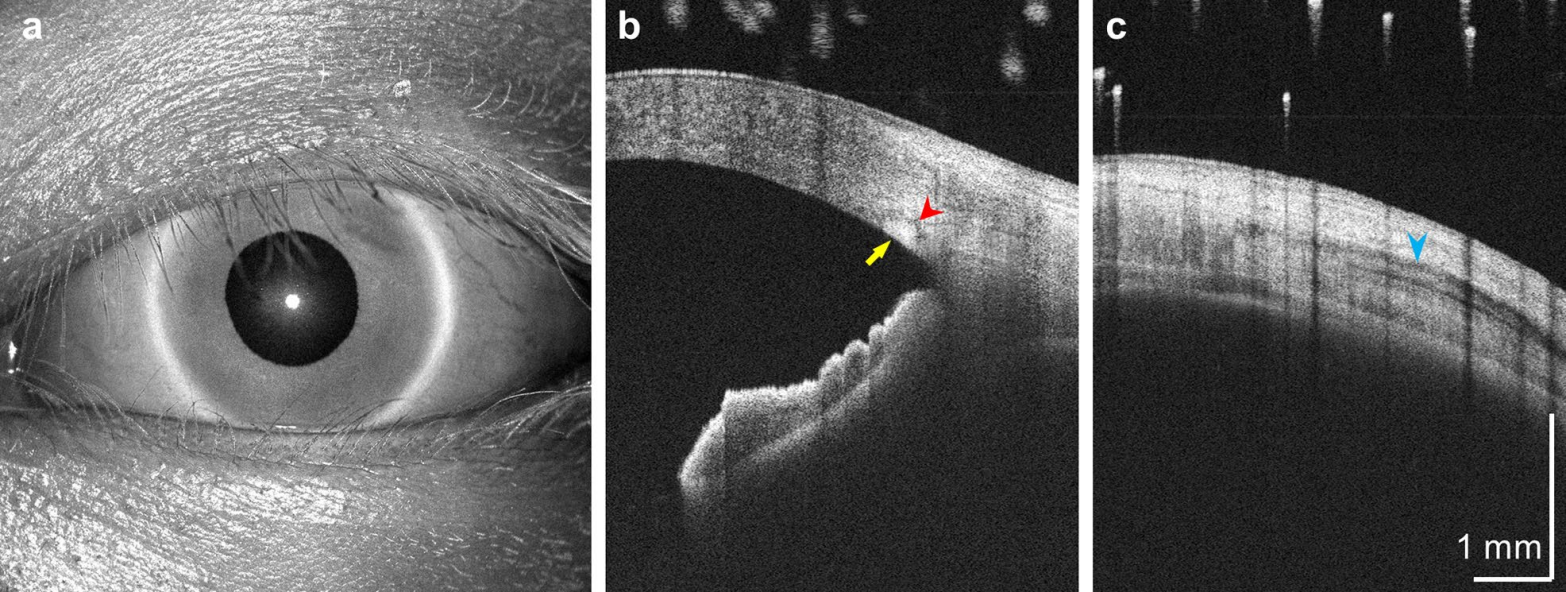
Wide field, long range, deep penetration structural imaging using the SS-OCT prototype instrument. (**a**) *En face* amplitude projection of a 25-mm OCT volume. (**b**) 6-mm horizontal B-scan centered at the limbus. Yellow arrow: trabecular meshwork. Red arrowhead: Schlemm’s canal. The connected hypo-reflective curvilinear feature may correspond to a collector vessel segment. (**c**) 6-mm horizontal B-scan 6 mm temporal to the corneoscleral junction. Blue arrowhead: insertion tendon of the lateral rectus muscle.

The 1310 nm wavelength band has low optical attenuation and scattering in tissue. The unique combination of deep penetration, long range, dynamic focusing, and ultrahigh imaging speed enables new imaging protocols for clinical and research applications. Figure 4a shows a wide field, volumetric scan over a 25-mm field of view (scan protocols described in the Methods section). This wide field of view volume scan can cover the entire anterior surface of the eye, while the acquisition time of 3.9 s minimizes motion artifacts. Figure 4b,c show B-scans centered at the corneoscleral junction and ~ 6 mm temporal to the junction, extracted from an OCTA scan protocol covering a 6 × 6 mm^2^ field of view and averaged 5 times. The deep image penetration using long wavelengths can clearly visualize subsurface features including the trabecular meshwork, Schlemm’s canal, and the lateral rectus muscle tendon insertion.

**Figure 5:**
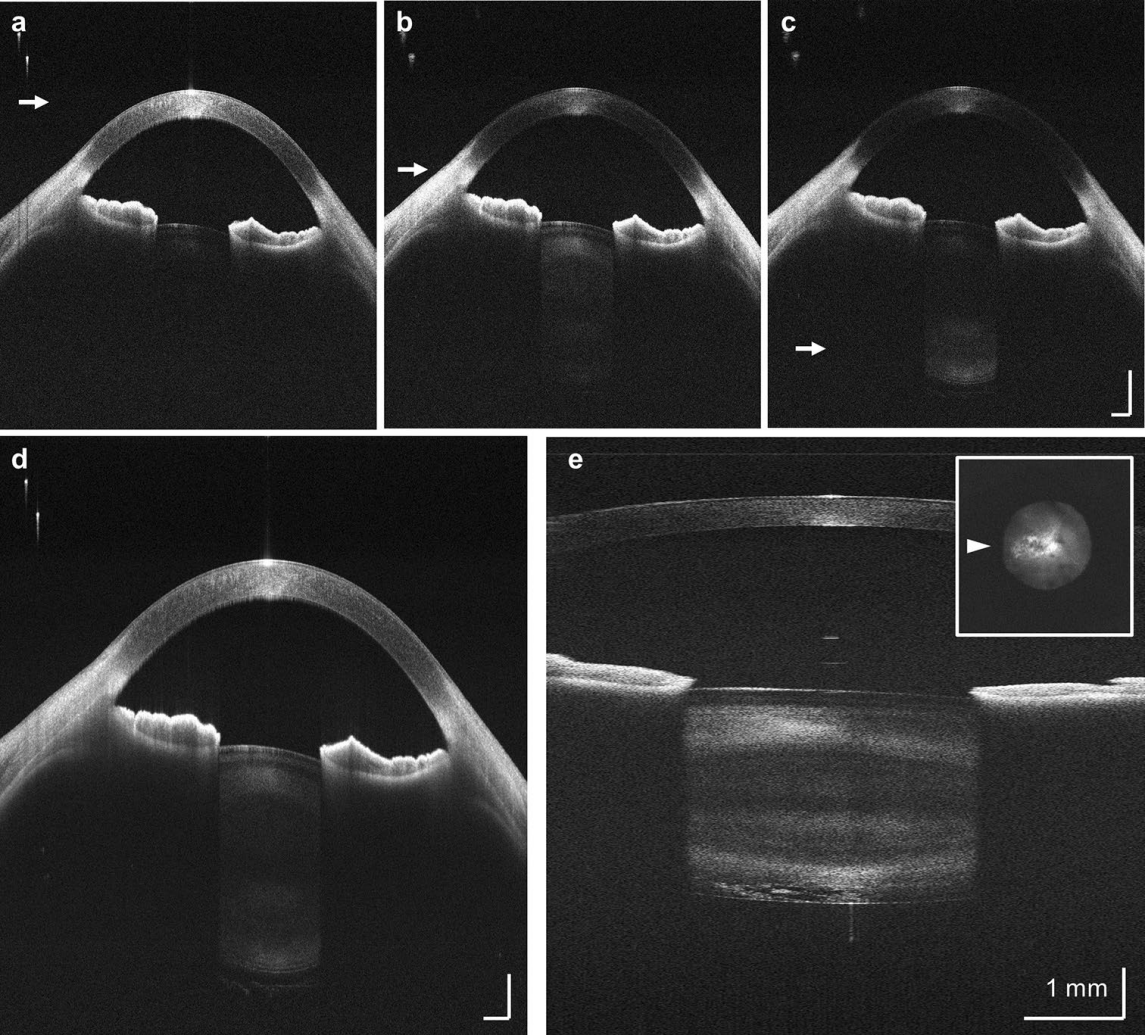
SS-OCT imaging of the full anterior segment of the eye. (**a**)–(**c**) Extracted 20-mm B-scans where the focal depth position is dynamically adjusted with 18 steps at ~ 500 µm spacing. The focal plane shifts from the cornea to the posterior surface of the lens capsule. White arrow: corresponding location of the focal plane. (**d**) Fused full depth high resolution cross-sectional image using all focally stepped B-scans. (**e**) 6-mm OCT volume of an eye with subcapsular cataract. The focal plane is fixed at the posterior lens capsule. The B-scan corresponds to the horizontal meridian (white arrowhead). Insert: *en face* OCT amplitude projection along the depth range of the posterior half of the lens capsule. The lateral extent of the cataract lesion can be appreciated. Saccadic motion was manually corrected using rigid strip based registration.

Dynamically adjusting the OCT beam focal plane position during the acquisition extends the effective depth of field to enable long range imaging without trading off transverse resolution. The focal plane position can be continuously stepped between repeated B-scans, shifting from the anterior surface of the cornea to the posterior surface of the lens capsule (Fig. 5a–c; Supplementary Video V2). As the focal plane is shifted between B-scans, different features, i.e., internal structure of the cornea, iris, and the lens capsule, are sequentially highlighted. A fused image can be generated from these B-scans using Gabor filtering^23^, providing simultaneous visualization of all corneal lenticular features within a single cross-sectional image (Fig. 5d).

Alternatively, the focal plane can be set to highlight a specific pathology. For example, Fig. 5e shows a central 6 mm B-scan highlighting a subcapsular cataract. The transverse extent of the cataract can be quantified by projecting the OCT signal amplitude along the depth range covering the posterior half of the lens, forming an *en face* view.

### OCT angiography of the anterior eye

The A-scan rate of 325 kHz represents a 2–5 × improvement over commercially available anterior eye instruments. This advance is especially important for OCTA imaging. OCTA uses multiple repeated B-scans to detect motion contrast from flowing blood cells and requires higher speed than structural OCT in order to image a clinically useful field of view with sufficient sampling density. The deep penetration of the 1310 nm wavelength also enables visualizing vascular contrast in highly scattering tissues such as the sclera and iris, and determining their depth profile.

**Figure 6:**
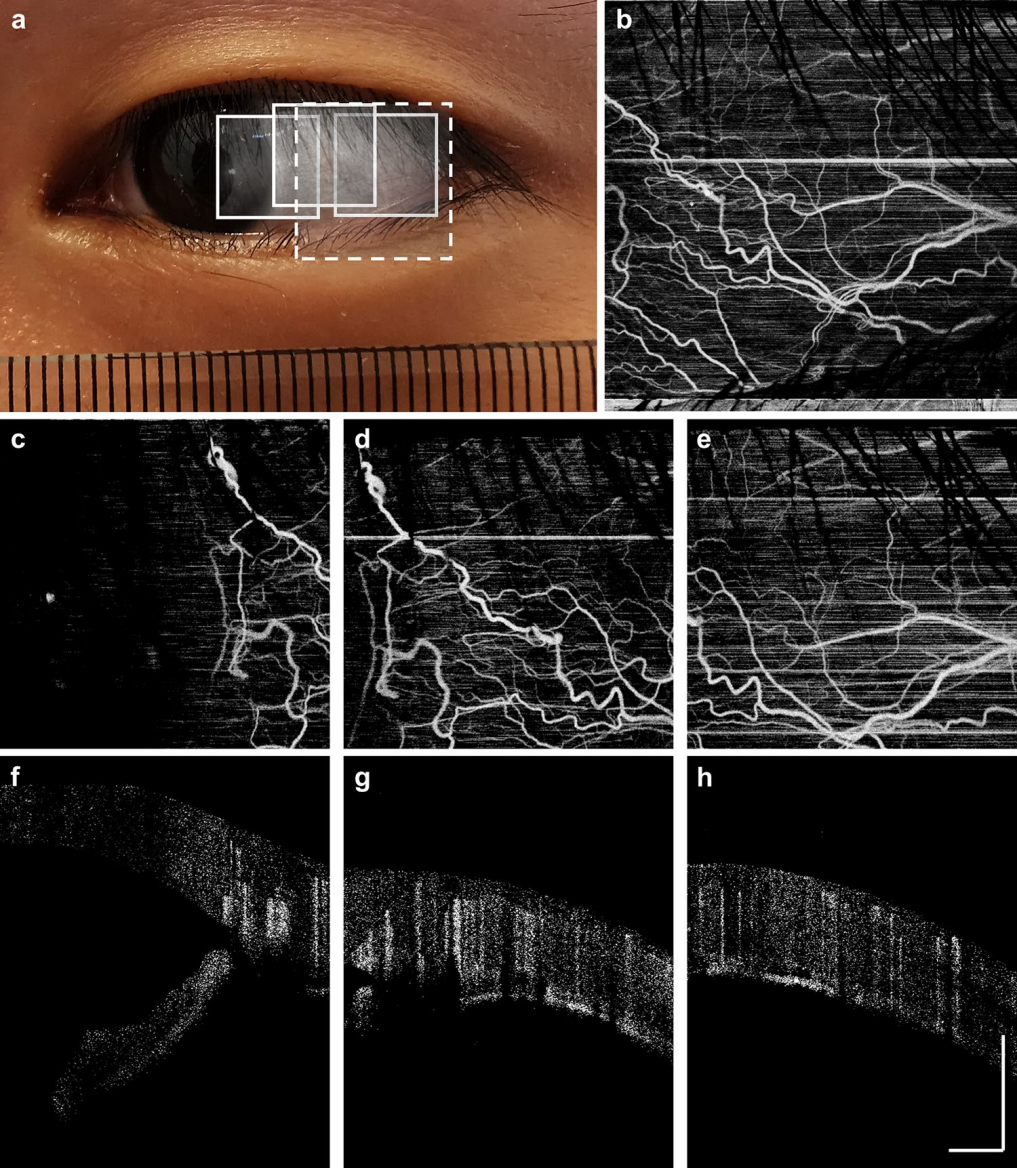
OCTA imaging using the SS-OCT prototype instrument. (**a**) Digital image of the subject. Boxes indicates locations of the OCTA scans. (**b**) 9-mm OCTA projection, scanned at the location corresponding to the dashed box. (**c**)–(**e**) 6-mm OCTA projections, scanned at locations corresponding to the solid boxes, respectively. (**f**)–(**g**) OCTA B-scans, extracted respectively from the center of the imaging field. Note the highly scattering sclera generates strong vertical projection artifacts. The OCTA signal posterior to the sclera is assumed to originate from the choroid.

Figure 6 shows OCTA mosaics from a healthy subject. We demonstrate OCTA with 6 × 6 mm^2^ and 9 × 9 mm^2^ fields of view (scan protocols described in the Methods section). In addition to showing vascular morphology in the *en face* projection, OCTA is inherently depth-resolved and can be used to differentiate conjunctiva and episcleral vessels using the OCTA B-scans. In the more temporal image, the choroid layer, which is rich in the vasculature, can also be seen on the interior aspect of the sclera (Fig. 6g,h).

**Figure 7:**
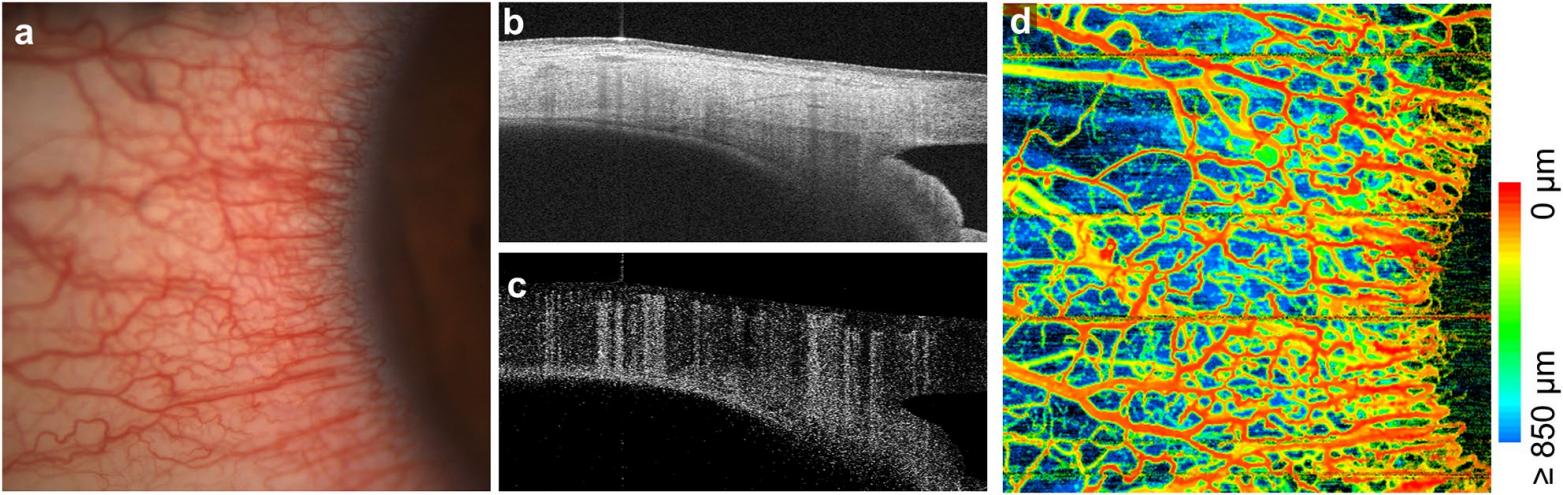
Increased ocular surface vessel density due to contact lens wear. (**a**) Color photography from a slit lamp examination. (**b**) Structural OCT B-scan from the center field of view. (**c**) Corresponding OCTA B-scan. (**d**) Depth resolved OCTA which is color coded by the relative vessel depth beneath the anterior conjunctiva surface.

Ocular surface OCTA is of clinical interest for monitoring vascular response and vessel remodeling. Changes in vascular morphology often occur in ocular surface inflammation, such as dry eye disease or contact lens wear. As an example, Fig. 7 shows a slit lamp photograph and 6 × 6 mm^2^ scleral OCTA from a contact lens wearer, revealing elevated vessel density. Comparing the depth map and B-scans reveals that most of the OCTA signal originates from shallower conjunctival or episcleral vessels, likely perfused or dilated in response to contact lens wear. Clinically, determining the affected vascular layers may be important for the differential diagnosis of ocular surface inflammation^24,25^.

**Figure 8:**
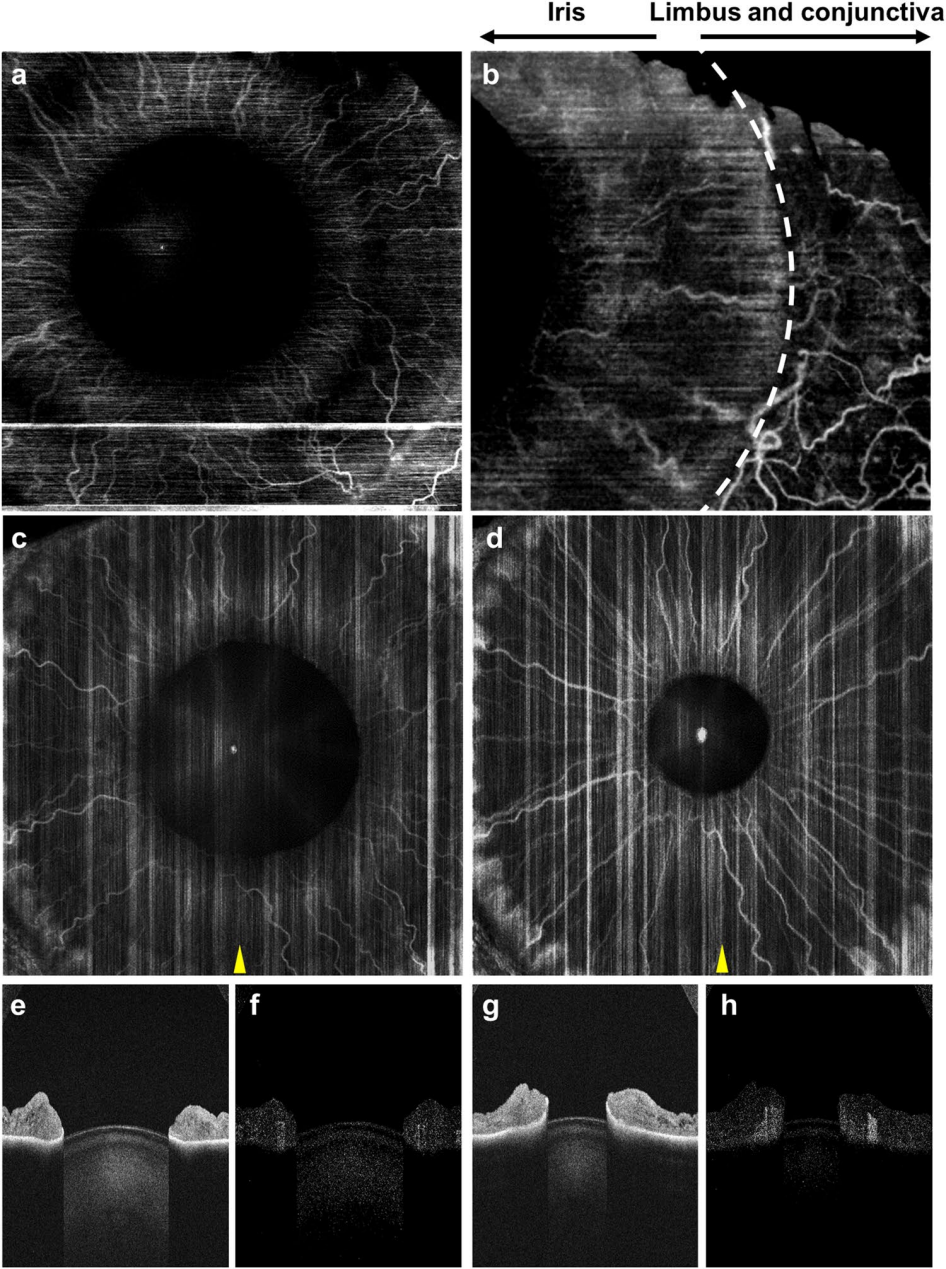
Iris OCTA using the SS-OCT prototype instrument. (**a**) 9-mm and (**b**) 6-mm OCTA of an Asian eye (dark pigmented). (**c**)–(**d**) 12-mm OCTA of a Caucasian eye (lightly pigmented). (**c**) shows a dilated pupil in dark environment, where (**d**) shows a constricted pupil in response to white light stimulation. Iris vessels show morphological changes following pupillary constriction, where the vessels appear straighter and brighter. (**e**, **g**) Structural and (**f**, **h**) angiographic B-scans extracted from the corresponding scans at the locations indicated by the yellow arrowhead.

Finally, we demonstrate OCTA imaging of the iris. Figure 8a,b show OCTA of a highly pigmented iris. Dark iris pigmentation attenuates the OCT beam, presenting a challenge for OCTA imaging. With reduced tissue scattering and increased penetration, 1310 nm wavelength SS-OCT has sufficient depth and contrast to resolve iris vasculature, revealing its unique radial arrangement. In comparison, a lightly pigmented iris was also imaged (Fig. 8c–h), where we induced pupillary constriction by using white light stimulation. OCT B-scans show morphological alterations of the iris following pupillary constriction, with an overall decrease in thickness. The vessels stretch and become straighter in the constricted iris. The OCTA signal becomes higher in the constricted iris. While not quantitatively verified in our study, the increased OCTA signal might be attributed to increased blood flow. However, the decrease of light absorption and scattering when the iris is constricted and thinner may also cause an increased OCTA signal.

## Discussion

The anterior segment occupies roughly the frontal third of the human eye and anatomically consists of the cornea, iris, and lens. Anterior eye imaging also extends to the limbus, conjunctiva, and sclera. The ability to image both structural and vascular features volumetrically and with high spatial resolution is essential for fundamental research and clinical studies. Applications include ocular surface evaluation and biometry, examination of the lens for cataracts and planning intraocular lens surgery, assessing the anterior angle and aqueous outflow for glaucoma diagnosis and monitoring, and investigating vessel morphology changes due to normal physiological response, inflammation, or tumor growth^9,26^.

The anterior eye is directly exposed to the environment and is located much shallower than the retina, which is posterior to the vitreous humor. These properties enable the use of the 1310 nm wavelengths, which are not applicable for retinal imaging due to high water absorption in the vitreous^27^. However, anterior eye imaging also has unique challenges. As mentioned earlier, imaging the anterior eye requires a depth range of more than 8 mm in tissue. In addition, the high optical scattering and attenuation of the sclera and iris also make deep tissue imaging challenging. These constraints have resulted in commercial anterior eye OCT instruments (Zeiss Visante; Tomey Casia 2; Heidelberg Engineering Anterion) being slower, lower resolution, or having limited functionality (e.g., lack of OCTA) compared to retinal OCT (Table 1). Retinal OCT instruments can be adapted for anterior eye imaging using accessory lens attachments (Optovue Angiovue; Zeiss Cirrus; Topcon Triton). However, these instruments are not optimized for anterior eye imaging. Specifically, Angiovue and Cirrus are spectral-domain OCT (SD-OCT) using 840 nm wavelengths with limited image penetration in the sclera or iris. SD-OCT also has a short imaging range and variation of sensitivity with range.

**Table 1:** Comparison of selected commercial OCT instruments and the SS-OCT prototype instrument. Axial resolution and imaging depth reported correspond to values in tissue.TD-OCT = time domain OCT.

Device	Type	Wavelength (nm)	A-scan rate (kHz)	Axial resolution (µm)	Imaging depth (mm)	Max. B-scan length (mm)	OCTA
Visante (Carl Zeiss Meditech)	TD-OCT	1310	2	18	6	16	No
Casia 2 (Tomey)	SS-OCT	1310	50	10	13	16	No
Anterion (Heidelberg Engineering)	SS-OCT	1300	50	10	14	~ 16	No
Angiovue (Optovue)*^1^	SD-OCT	840	70	5	Up to 3	~ 8	Yes
Cirrus (Carl Zeiss Meditech)*^1^	SD-OCT	840	68	5	Up to 2.9	15.5	Yes
Triton (Topcon)*^1^	SS-OCT	1050	100	8	~ 3.0	16	Yes
Prototype	SS-OCT	1310	325	9	11.6	25	Yes

*^1^Retinal OCT instruments. Imaging the anterior eye is possible through the use of accessory lens.

The prototype instrument described in this study incorporates advances in SS-OCT technology that enhance structural and angiographic imaging of the anterior eye and are translatable to multiple applications. Anterior eye OCT instruments with rapid A-scan rate, long range, or full eye imaging capability were previously demonstrated with individual functionality^28–30^. However, to our knowledge, this is the first demonstration of simultaneous high speed (325 kHz), long range (up to 15.5 mm in air), wide field OCT and OCTA with deep image penetration and dynamic focusing in an integrated instrument. This unique capability is enabled by combining an optimized MEMS-VCSEL light source, linearized *k* sweep, high speed dual channel OCT and MZI acquisition, sweep to sweep *k* calibration, dynamic focusing, and stable and ergonomic patient interface design.

The design parameters represent a balance between speed, resolution, and imaging range, chosen to address imaging requirements of a wide range of clinical applications, including intraocular lens power calculation, sclera lens fitting, and investigating microvascular alterations in the anterior eye. Accurate biometry and intraocular lens power calculation using appropriate formulas are essential to reducing refractive error after cataract surgery^31,32^. Most intraocular lens formulas estimate parameters such as anterior chamber depth and equatorial lens position using regression modeling, mainly because direct measurements are unavailable or challenging^33,34^. As a result, unsatisfactory refractive outcomes are common in eyes with corneal irregularity or prior refractive surgery. The long range and high speed imaging capability of SS-OCT technology should facilitate biometry of all optical surfaces from the anterior cornea to the posterior lens, which has been shown to improve lens power calculation in recent pilot studies^31,35^.

Our group pioneered the use of OCT to measure the meridional scleral sagittal depth to aid sclera lens fitting^36^. This method is being adopted to replace impression molding, reduce or eliminate the initial trial and error process of sclera lens selection, and assess the settling and quantify ocular response to lens wear^37^. The large field of view and high imaging speed of prototype SS-OCT technology also enables full volumetric assessment of corneoscleral curvature, which does not rely on circular symmetry and can provide comprehensive evaluation of lens conformity and fit with high spatial resolution and accuracy.

The application of OCTA to investigate vessel morphology has been demonstrated for monitoring inflammation^38^, corneal neovascularization^39,40^, limbal stem cell deficiency^41^, and tumor growth^42^. Thus, anterior eye OCTA may serve as a non-invasive alternative to fluorescein angiography for assessing vascular morphology to determine malignancy. For example, elevated vessel density and/or tortuosity may be markers of malignancy in iris tumors^43^. These objective markers can complement traditional markers such as size, thickness, growth rate, and/or seeding, offering both the ophthalmologist and patient an informed decision for treatment strategy, which can improve survival and preserve vision.

This study presents pilot imaging results on normal anatomical features and selected examples of pathologies, including cataracts and elevated vessel density due to contact lens wear. The scan protocols in this study primarily serve as a proof-of-concept to demonstrate the imaging capability of the SS-OCT prototype. Although these are selected examples, we believe they demonstrate the potential for investigating many different anterior eye diseases. Accordingly, imaging protocols targeting specific pathologies will require careful design and optimization. For example, the scanning protocol can be adapted to accommodate features such as the iris, anterior chamber angle, or lens to reduce acquisition time and/or improve coverage. In this study, we used a fixed focal plane for the raster and OCTA protocols. However, it is possible to estimate the depths of ocular surfaces from pilot scans, and dynamically adjust the focal plane accordingly. This will compensate the sclera curvature for large field of view OCTA, as well as enable simultaneous high-resolution biometry of the cornea and sclera.

It is interesting to note that the deep penetration of 1310 nm wavelength light and the OCT imaging performance of the prototype instrument allows visualization of the choroid from the anterior scleral aspect. Currently, there are limited reports of optical transscleral assessment of the choroid^44^, and OCTA contrast has not been previously demonstrated. While the clinical utility remains unclear, demonstrating this capability can inform the community and encourage further investigation.

A limitation of the study is that we mainly imaged healthy subjects without pathologies. Thus, we cannot explicitly demonstrate that the technology can improve clinical imaging performance in diseased eyes. However, the prototype instrument achieves a combined imaging speed and range not shown previously, with increased field of view, functionality, and stability. These advancements should contribute to improved imaging performance as well as higher quality and yield, which would facilitate both qualitative and quantitative diagnostic metrics. In this study, we did not correct for artifacts caused by eye motion or OCTA projection artifacts. However, many correction algorithms from retinal imaging should apply to the anterior eye^45–48^. We believe that a variety of new software developments designed for the optics, geometry, and motion of the anterior eye will enable a wide range of structural and functional measurements. The performance of the prototype instrument is limited by the availability of high speed commercial A/D converters rather than the MEMS-VCSEL light source or imaging sensitivity. Future technological advancements in A/D converters should enable even faster A-scan rates and longer imaging ranges.

## Methods

### Anterior eye SS-OCT prototype instrument design

**Figure 9:**
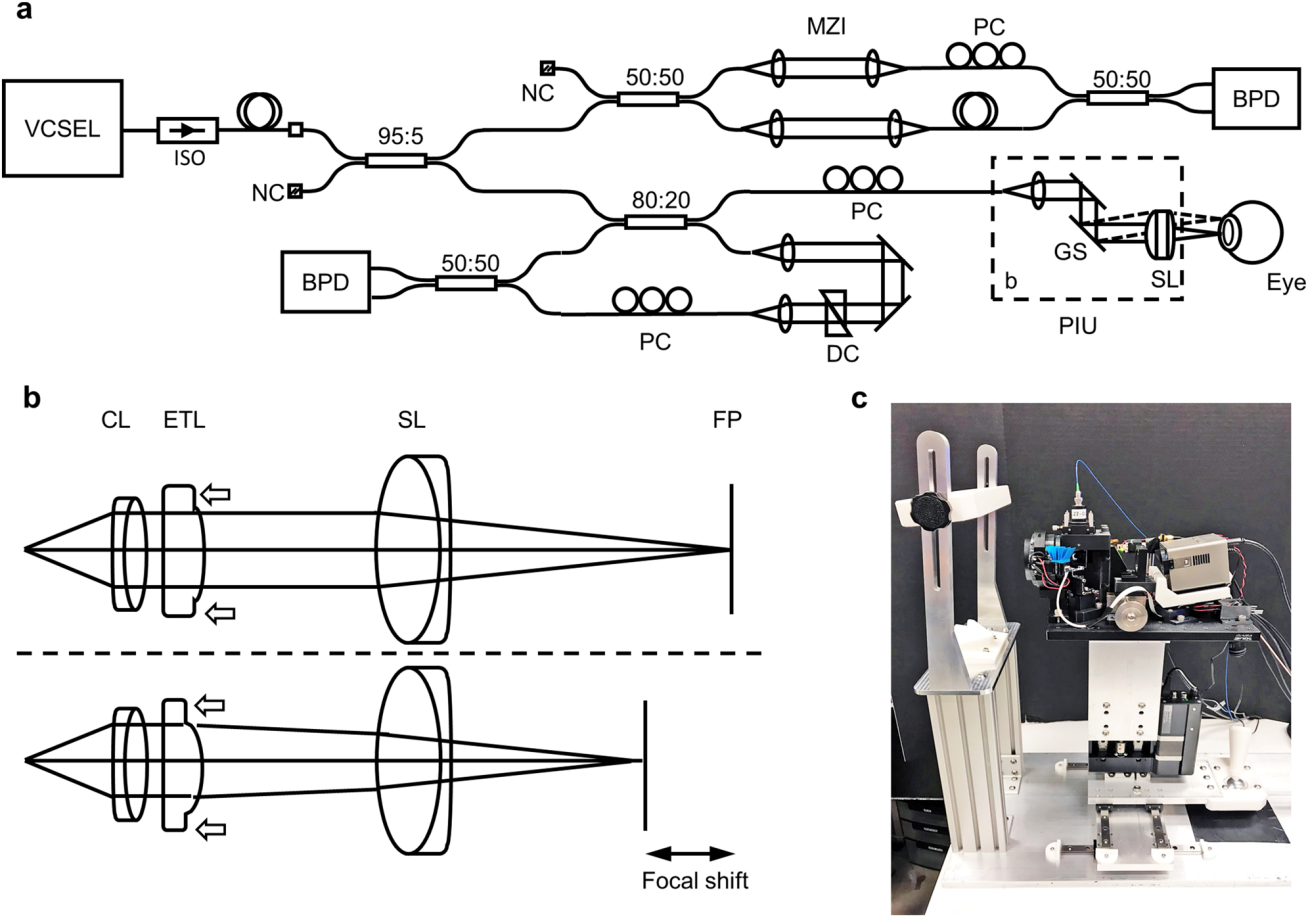
SS-OCT prototype instrument design. (**a**) Schematic of the high speed SS-OCT interferometer. BPD: balanced photo detector; DC: dispersion compensation glass plates; GS: galvanometer scanner; ISO: optical isolator; MZI: Mach–Zehnder interferometer; NC: not connected; PC: polarization controller; PIU: patient interface unit; SL: scan lens. (**b**) Simplified diagram showing dynamic focal plane adjustment using an electrically focus tunable lens. CL: collimating lens; ETL: electrically tunable lens; FP: focal plane. (**c**) Photo of the OCT patient interface unit with cover removed.

The prototype instrument utilizes recent advances in high-performance SS-OCT technology (Fig. 9a)^13^. The MEMS-VCSEL light source was optimized using a custom designed MEMS voltage drive waveform to generate a linearized frequency/wavenumber (*k*) versus time sweep in order to efficiently utilize A/D bandwidth. The sweep repetition rate was set to 325 kHz, optimizing the speed, sensitivity, and depth range for anterior eye imaging. Only the forward sweep (long-to-short wavelength sweep) is used for imaging in order to avoid variations in PSF, which can occur when bi-directional sweeps are used. The laser output is modulated off during backward sweep and the overall duty cycle is ~ 65%. The MEMS-VCSEL output is split by a 2 × 2 fiber coupler, respectively delivered to the OCT interferometer and the calibration MZI. The total fiber length is matched between the two interferometers to minimize dispersive phase distortion. Interference fringes are detected using 1.6 GHz bandwidth dual balanced detectors (PDB480C-AC, Thorlabs Inc.) and digitized at 2 Giga samples per second (ATS9373, Alazar Technologies Inc.).

In addition to 2-axis X/Y galvanometer scanners (6215H, Cambridge Technology), the patient interface unit incorporates an electrically focus tunable lens (EL-16-40-TC-NIR-20D, Optotune AG) to perform dynamic focusing^14,15^. The tunable lens power can be adjusted between − 10 and 10 diopters and is placed next to a fixed focal length achromatic collimator (focal length *f* = 19 mm, Edmund Optics Inc.). The resultant beam diverges or converges slightly before being focused by the achromat scan lens (effective *f* = 60 mm, Edmund Optics Inc.), shifting the focal plane posteriorly or anteriorly (Fig. 9b). The full 20 diopter range corresponds to ~ 71 mm focal shift; however, the usable range is limited by the clear aperture of the scanning optics and the OCT depth imaging range. For small focal steps of ~ 0.5 mm, the response and settling time is approximately 4 ms.

Two miniature CMOS cameras (MU181CR-ON-FL, Ximea GmbH) with *f* = 20 mm lenses (Edmund Optics Inc.), located on the sides of the OCT scan lens, provide a parallax “split view” of the iris to facilitate alignment. Similar implementations were also used on commercial instruments (e.g., Topcon Maestro). The angle between the camera axes and the OCT scan lens axis is +/− 30°. The digital images from the two camera views are vertically divided into two halves and stitched digitally. The composite image of the iris appears centered and appears whole only when both transverse and axial alignment is achieved. The image appears sheared if the axial alignment (instrument to pupil distance) is either too close or too far (Supplementary Video V1). Optically, each camera pixel (pixel pitch 1.25 µm) corresponds to ~ 8.8 µm lateral or ~ 15.2 µm axial displacement around the target position.

We also improved the stability and ergonomics of the patient interface using extruded / CNC machined aluminum and rapid prototyping (3D printing) (Fig. 9c). Dual stainless steel rails provide rigidity and stability for both the left–right and forward–backward translations. A high load, motorized stage (X-LRQ075HP-E01, Zaber Technologies Inc.) provides micrometer-precision vertical adjustment. The forehead rest and chin rest both have center V-groves that mitigate lateral translation, minimizing head tilt and rotation during the scan. The patient interface has an approximately 5° forward inclination, encouraging contact with the forehead rest during imaging. This further reduces patient head motion.

### Human subject recruitment and imaging protocols

The Institutional Review Board (IRB) / Committee on the Use of Humans as Experimental Subjects (COUHES) at Massachusetts Institute of Technology (MIT) approved the study protocol. All experimental procedures adhered to the tenets of the Declaration of Helsinki and complied with the Health Insurance Portability and Accountability Act of 1996. Subjects were recruited at the MIT campus and written informed consent was obtained prior to imaging.

A series of scan protocols were developed (Table 2) which utilized the unique capabilities of the SS-OCT prototype instrument, i.e., long image range, high speed, and adjustable focus. The example protocols are generalized from existing anterior eye instruments, as well as adapted from retinal OCT and OCTA. The high speed enables wide field, dense data sets to be acquired with short acquisition times. Each subject was imaged using one or more protocols and repeat acquisition was performed as required. Each imaging session lasted less than 30 min.

**Table 2:** OCT and OCTA scan protocols used in this study. The order of the A-scan numbers is: [Number of A-scans per B-scan] × [Number of B-scan repeats (OCTA only)] × [Number of B-scan locations per volume (volumetric scans only)].

Protocols	Field of view	Number of A-scans	Focus	Acquisition time
Linear scan*^1^	20 mm	1600	Fixed or stepped*^2^	5.0 ms
15-mm raster	15 × 15 mm^2^	800 × 800	Fixed	2.6 s
25-mm raster	25 × 25 mm^2^	1000 × 1000	Fixed	3.9 s
6-mm OCTA*^3^	6 × 6 mm^2^	400 × 4 × 400	Fixed	2.6 s
9-mm OCTA*^3^	9 × 9 mm^2^	500 × 4 × 500	Fixed	3.9 s
12-mm OCTA*^3^	12 × 12 mm^2^	500 × 4 × 500	Fixed	3.9 s

*^1^Multiple linear scans can be repeated, arranged in a radial scan pattern, or a combination.

*^2^The focal plane can be kept constant, or stepped, i.e. from the anterior corneal surface to the posterior surface of the lens capsule, between successive linear scans.

*^3^Structural OCT can also be generated by simple averaging of repeated B-scans in order to improve signal-to-noise.

### OCT and OCTA reconstruction

SS-OCT requires *k* calibration before the Fourier transform of the interference fringes can yield depth resolved A-scans. Therefore, accurate sweep calibration is critical to maintain sensitivity and constant axial resolution for accurate depth measurements over a long imaging range. Most SS-OCT instruments use optically clocked A/D conversion to avoid computationally resampling the OCT interference fringe^49,50^. The clock signal is typically derived from a calibration MZI, so the sampling is inherently linear in *k*. However, optical clocking has a limited A/D rate, and the achievable sampling rate is only ~ 1.3 GHz when using a 2 GHz digitizer^13^. Alternatively, the instantaneous phase of the laser sweep can be computationally calculated from the interference fringe of the calibration MZI. Recording both OCT and MZI interference fringes, and using the MZI for sweep calibration, have been demonstrated to achieve high phase stability^51–53^. However, the process is computationally intensive, especially for the high speed and large number of A-scans in our imaging protocols.

To address this challenge, we utilized modern general-purpose graphics processing units (GPU). Using parallel computing techniques, the MZI calibration fringe is bandpass filtered, and the instantaneous phase is retrieved from the Hilbert transform of the resultant analytical signal. Fiducial markers in the MEMS-VCSEL sweep (i.e., the sweep reversal reflex and the spectral shape) are used to resolve the 2π ambiguity^13^. The internal circuitry of the MEMS-VCSEL source monitors and stabilizes the wavelength sweeping range. Thus, the fiducial markers represent fixed wavenumber references for phase retrieval. The MZI fringe is empirically set to about 20% of the Nyquist frequency, or 200 MHz, which provides good calibration accuracy. Importantly, capturing the entire calibration sweep enables compensation of the nonlinear phase and amplitude response in the detectors and electronics^11^. Group delay dispersion is corrected using a phase vector. Unless otherwise noted, a Taylor window with 30 dB sidelobe suppression is applied to reduce high signal artifacts from brighter surfaces. Finally, inverse Fourier transform is used to reconstruct the complex OCT signal.

After sweep to sweep *k* calibration and OCT reconstruction, the structural (amplitude) OCT images are generated from the absolute value of the reconstructed complex signal. If repeated B-scans are acquired, such as in OCTA protocols, rigid image registration^54^ and arithmetic frame averaging enhance the signal-to-noise ratio. OCTA is calculated using an amplitude decorrelation algorithm^55^, where all B-scan pairs (e.g., 1 ↔ 2, 2 ↔ 3, …; 1 ↔ 3, 2 ↔ 4; …) are included. For ocular surface OCTA, a segmentation algorithm based on edge-detection is used to identify the anterior conjunctiva surface. As a result, the OCTA projection includes only axial ranges within the conjunctiva and sclera (0 µm to ~ 500–600 µm below the surface, dependent on eye and sclera curvature). This improves OCTA contrast by excluding choroidal vessels in the en face OCTA visualization.

## Supplementary Information

Supplementary Information 1.

Supplementary Video 1.

Supplementary Video 2.

## Data Availability

The instrument design, data sets and algorithms from this study are available from the corresponding author upon reasonable request.
