# Supplementary material for: High speed, long range, deep penetration swept source OCT for structural and angiographic imaging of the anterior eye
Source: Sci Rep. 2022 Jan 19;12:992. doi: 10.1038/s41598-022-04784-0 (PMC8770693; doi:10.1038/s41598-022-04784-0)
Supplement: Supplementary file 1 — Supplementary Information 1. [file 41598_2022_4784_MOESM1_ESM.docx]

# Supplementary Videos

**Video V1.** Aiming the instrument using the split view iris cameras. Two miniature digital cameras, one on each side of the scan lens, provide a pallax view of the iris to facilitate aligning the instrument to subjet’s eye. Iterative adjustments center and match the top and bottom halves of the iris image. The iris appears sheared if the axial alignment (instrument to pupil distance) is either too close or too far. The image of the iris appears centered and appears whole only when both transverse and axial alignment are achieved.

**Video V2.** Radial meridian scan with inter-B-scan focal sweep. Each 20-mm meridional view consists of 18 B-scans with stepped focus from the anterior corneal surface to the posterior lens capsule ~500 µm apart. Dynamically adjusting the focal plane during the acqsuition extends the effective depth of field to enable long range imaging without sacrificing transverse resolution. Video playback is at 10× slowdown of the scanning speed.
